# Gene expression analysis reveals that Delta/Notch signalling is not involved in onychophoran segmentation

**DOI:** 10.1007/s00427-016-0529-4

**Published:** 2016-03-02

**Authors:** Ralf Janssen, Graham E. Budd

**Affiliations:** Department of Earth Sciences, Palaeobiology, Uppsala University, Villavägen 16, 75236 Uppsala, Sweden

**Keywords:** Onychophora, Segmentation, Delta, Notch, Posterior elongation

## Abstract

Delta/Notch (Dl/N) signalling is involved in the gene regulatory network underlying the segmentation process in vertebrates and possibly also in annelids and arthropods, leading to the hypothesis that segmentation may have evolved in the last common ancestor of bilaterian animals. Because of seemingly contradicting results within the well-studied arthropods, however, the role and origin of Dl/N signalling in segmentation generally is still unclear. In this study, we investigate core components of Dl/N signalling by means of gene expression analysis in the onychophoran *Euperipatoides kanangrensis*, a close relative to the arthropods. We find that neither *Delta* or *Notch* nor any other investigated components of its signalling pathway are likely to be involved in segment addition in onychophorans. We instead suggest that Dl/N signalling may be involved in posterior elongation, another conserved function of these genes. We suggest further that the posterior elongation network, rather than classic Dl/N signalling, may be in the control of the highly conserved segment polarity gene network and the lower-level pair-rule gene network in onychophorans. Consequently, we believe that the pair-rule gene network and its interaction with Dl/N signalling may have evolved within the arthropod lineage and that Dl/N signalling has thus likely been recruited independently for segment addition in different phyla.

**Electronic supplementary material:**

The online version of this article (doi:10.1007/s00427-016-0529-4) contains supplementary material, which is available to authorized users.

## Introduction

One of the key features of the arthropods is their segmented body. Most knowledge about the molecular mechanisms underlying the arthropod segmentation process, however, comes from a single model organism, the fly *Drosophila melanogaster*. In *Drosophila*, the body becomes segmented more or less simultaneously during development. The quick subdivision of the embryonic body into smaller and smaller units and finally the segments (or parasegments) is achieved by the function of the so-called segmentation genes (Akam 1987; Pick 1998; Sanson 2001). The long-germ developmental mode by which the *Drosophila* embryo is patterned, however, is highly derived (e.g. Liu and Kaufman 2005; Kimelman and Martin 2012). The great majority of arthropods, including most other insects, add segments one by one or in pairs from a posterior segmentation zone (Chipman et al. 2004; Schoppmeier and Damen 2005a; Janssen 2011, 2014)—so-called short-germ mode development. Most of the segmentation genes that act during *Drosophila* segmentation also play a conserved or similar role in the segmentation process in short-germ arthropods (e.g. Choe et al. 2006; Choe and Brown 2009; Damen 2002; Damen et al. 2000, 2005; Janssen et al. 2004, 2011a, b). It is therefore widely accepted that arthropod segmentation has evolved only once in the common ancestor of all arthropods, i.e. chelicerates, myriapods, crustaceans and insects (e.g. Peel et al. 2005; Damen 2007). Research on onychophorans, a closely related sister group to the arthropods (Campbell et al. 2011), is congruent with this idea and suggests that the common ancestor of onychophorans and arthropods also used a partially conserved genetic program to segment its body (Eriksson et al. 2009; Janssen and Budd 2013; Franke and Mayer 2014; Franke et al. 2015).

Several studies have demonstrated or suggested that Delta/Notch (Dl/N) signalling is an important component of the gene regulatory system that underlies segmentation in a wide range of short-germ arthropods including chelicerates (Stollewerk et al. 2003; Schoppmeier and Damen 2005b; Oda et al. 2007), myriapods (Dove and Stollewerk 2003; Kadner and Stollewerk 2004; Chipman and Akam 2008), crustaceans (Williams et al. 2012; Eriksson et al. 2013) and insects (Chesebro et al. 2012). Surprisingly, however, it appears that the involvement of Dl/N is not generally conserved (or at least needed) in arthropods to segment their bodies. It has been shown that Dl/N has lost its function in segmentation in the holometabolous insects to which *Drosophila* belongs (Wilson et al. 2010, but Liu 2013), while the role of Dl/N in hemimetabolous insects is still under discussion (Pueyo et al. 2008; Kainz et al. 2011; Mito et al. 2011). This means that Dl/N signalling was either a component of the ancestral arthropod segmentation mechanism or that it has been recruited several times independently within the arthropod lineages. Despite the still unresolved situation in arthropods, it has even been suggested that Dl/N signalling could be a part of a common and conserved segmentation mechanism in all segmented phyla and, thus, in a segmented bilaterian ancestor (e.g. Stollewerk et al. 2003; Pueyo et al. 2008). This is because Dl/N signalling is also an important component of segment (somite) addition in vertebrates and possibly also in annelids (e.g. Pourqui 2003; Rivera et al. 2005; Thamm and Seaver 2008).

Another conserved role of Dl/N signalling is its function during posterior elongation (e.g. Oda et al. 2007; Mito et al. 2011; Williams et al. 2012). It is assumed that Dl/N signalling may have been an ancestral bilaterian component of posterior elongation more generally than that in segmentation, a function that this gene regulator network may have evolved much later and independently in overtly segmented phyla such as arthropods (reviewed in Chipman 2010).

Expression patterns of onychophoran *Notch* and *Delta* genes have previously been described by Oliveira et al. (2013) for a closely related onychophoran, *Euperipatoides rowelli*, and by Eriksson and Stollewerk (2010) for *Euperipatoides kanangrensis*. The work of Oliveira et al. (2013) focuses on the development of muscle attachment sides and the analysis of gene expression is restricted to very late embryos. This paper is thus of little help to unravel a potential role of Notch signalling in segmentation. The work by Eriksson and Stollewerk (2010) does not focus on segmentation either, but on the development of the nervous system. Expression patterns that could be associated with a role in segmentation or posterior elongation have not been provided in this publication.

In order to elaborate hypotheses about the origin and evolution of Dl/N function during segmentation, it is thus necessary to investigate the expression patterns of key components of Dl/N signalling during segment addition, including the very earliest stages. We therefore studied the embryonic expression profiles of the transmembrane receptor Notch (N), its potential ligands Delta (Dl) and Serrate (Ser) and the Notch-dependent transducing transcription factor Suppressor of Hairless (Su(H)) in the onychophoran *E. kanangrensis*. If Dl/N signalling is a component of onychophoran segmentation, one would expect canonical Dl/N signalling factors to be expressed in the posterior segmentation zone and in newly formed segments, either ubiquitously or in distinct transverse stripes, as it is the case for these genes in arthropods (Dove and Stollewerk 2003; Kadner and Stollewerk 2004; Chipman and Akam 2008; Schoppmeier and Damen 2005b; Stollewerk et al. 2003). If, however, Dl/N signalling is merely involved in posterior elongation, another conserved function of these genes, then one would expect the expression in the posterior tip of the developing embryo.

## Methods

### Embryo collection, fixation and staging

Embryos were collected, fixed and stored for subsequent in situ hybridization experiments as described in Janssen et al. (2015a). Embryos were staged according to Janssen and Budd (2013).

### Gene cloning

Total RNA was isolated from *E. kanangrensis* embryos of different stages using TRIzol (Invitrogen). Poly-A RNA was extracted from total RNA (PolyATtract mRNA Isolation System III, Promega) and reversely transcribed into cDNA (SuperScript II First-Strand Synthesis System for RT-PCR, Invitrogen). All investigated gene fragments were isolated by means of PCR with gene-specific primers based on a sequenced embryonic transcriptome (Janssen and Budd 2013). In all cases, a first PCR was followed by a second (nested) PCR. Fragments were then cloned into pCR II vectors (TA Cloning Kit Dual Promoter; Invitrogen, Carlsbad, CA, USA). Sequences of isolated gene fragments were determined on a 3100 automated sequencer (Terminator Cycle Sequencing Kit; PerkinElmer Applied Biosystems, Foster City, CA, USA) using BigDye dye terminators version 3.1 (BigDye Terminator Cycle Sequencing Kit; PerkinElmer Applied Biosystems, Foster City, CA, USA). Gene sequences are available under accession numbers LN881709 (*Ek-N*), LN881710 (*Ek-Dl*), LN881711 (*Ek-Su(H)*) and LN881712 (*Ek-Ser*).

### Gene orthology

Identity of the isolated gene fragments was determined previously for Notch and Delta (Eriksson and Stollewerk 2010) (and for the closely related species *E. rowelli* (Oliveira et al. 2013)). The orthology of the second investigated Notch ligand, Ek-Ser, is proven by the presence of N-terminal N-terminus of Notch ligand (MNNL) and Delta-Serrate ligand (DSL) domains (both shared with Delta) and the Serrate-specific C-terminal von Willebrand domain type C (VWC) domain (Marchler-Bauer et al. 2015). The Suppressor of Hairless protein is unique since it contains LAG1, BTD and IPT domains (Marchler-Bauer et al. 2015). Overall, the sequence of this gene is highly conserved among bilaterian animals.

### Whole-mount in situ hybridization and nuclear staining

In situ hybridization was performed as described by Janssen et al. (2015a). Digoxigenin-labelled RNA probes were transcribed from the cloned fragments. *E. kanangrensis* embryos were hybridized with the probes at 62 °C for at least 16 h. No protein K treatment and no additional fixation were performed. Nucleic staining was performed by incubation of the embryos in 1 μg/ml of the fluorescent dye 4′,6-diamidino-2-phenylindole (DAPI) in phosphate-buffered saline with 0.1 % Tween-20 (PBST) for 40 min.

### Data documentation

Embryos were analysed under a Leica dissection microscope equipped with a Leica DC100 digital camera. The image processing software Adobe Photoshop CS2 (version 9.0.1 for Apple Macintosh) was used for linear corrections of brightness, contrast and colour values in all images.

## Results

### Expression patterns

Our study verifies most of the previously reported gene expression patterns of *Euperipatoides Notch* and *Delta* genes (Eriksson and Stollewerk 2010; Oliveira et al. 2013).

**Fig. 1:**
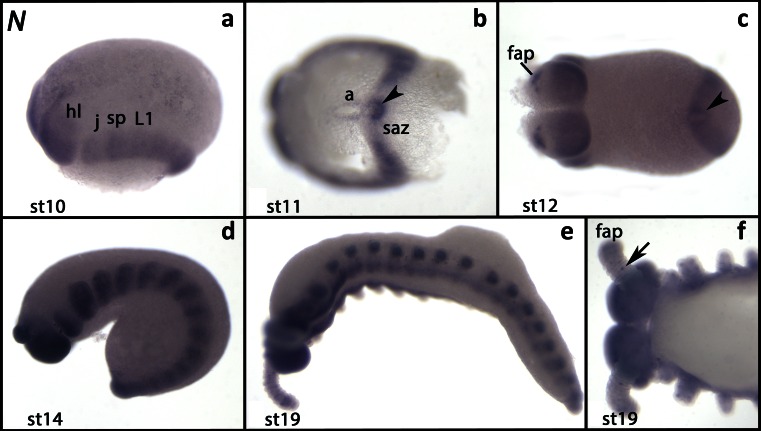
Expression of *Notch*. In all panels, the anterior is to the *left*. **a** Lateral view (stage 10). **b** Ventral view. The posterior end of the embryo is in focus. *Arrowhead* points to the expression in the tissue surrounding the posterior pit (stage 11). **c** Ventral view (stage 12). *Arrowhead* as in **b. d** Lateral view (stage 14). **e** Lateral view (stage 19). **f** Dorsal view of the anterior of the same embryo as shown in **e**. *Arrow* points to a single cell in the frontal appendage. *a* anus, *fap* frontal appendage, *j* jaw, *hl* head lobe, *L* walking limb, *saz* segment addition zone, *sp* slime papilla

In early developmental stages, *Euperipatoides Notch* (*Ek-N*) is expressed ubiquitously. Higher levels of expression, however, are in the posterior of the head lobes (compared to the anterior region) and in the posterior pit (Fig. S1A/B). Later, expression disappears from the anterior half of the head lobes and the segment addition zone (Figs. 1a, b and S1B). The posterior pit, however, still expresses *Ek-N* (Figs. 1b, c and S1B). In the anterior hemisphere of the head lobes, *Ek-N* is only expressed in few cells in the developing frontal appendages (Fig. 1c). We assume that these are antennal sense organs (cf. Mayer and Whitington 2009; Eriksson and Stollewerk 2010). In later developmental stages, *Ek-N* is strongly expressed in tissue ventral to the limbs and in the developing limbs (Fig. 1d, e). Expression in the frontal appendages and the trunk appendages is upregulated in single cells or small cell clusters (Fig. 1f). We do not detect a “transverse stripe in the ventral protocerebral primordium” (cf. Eriksson and Stollewerk 2010).

**Fig. 2:**
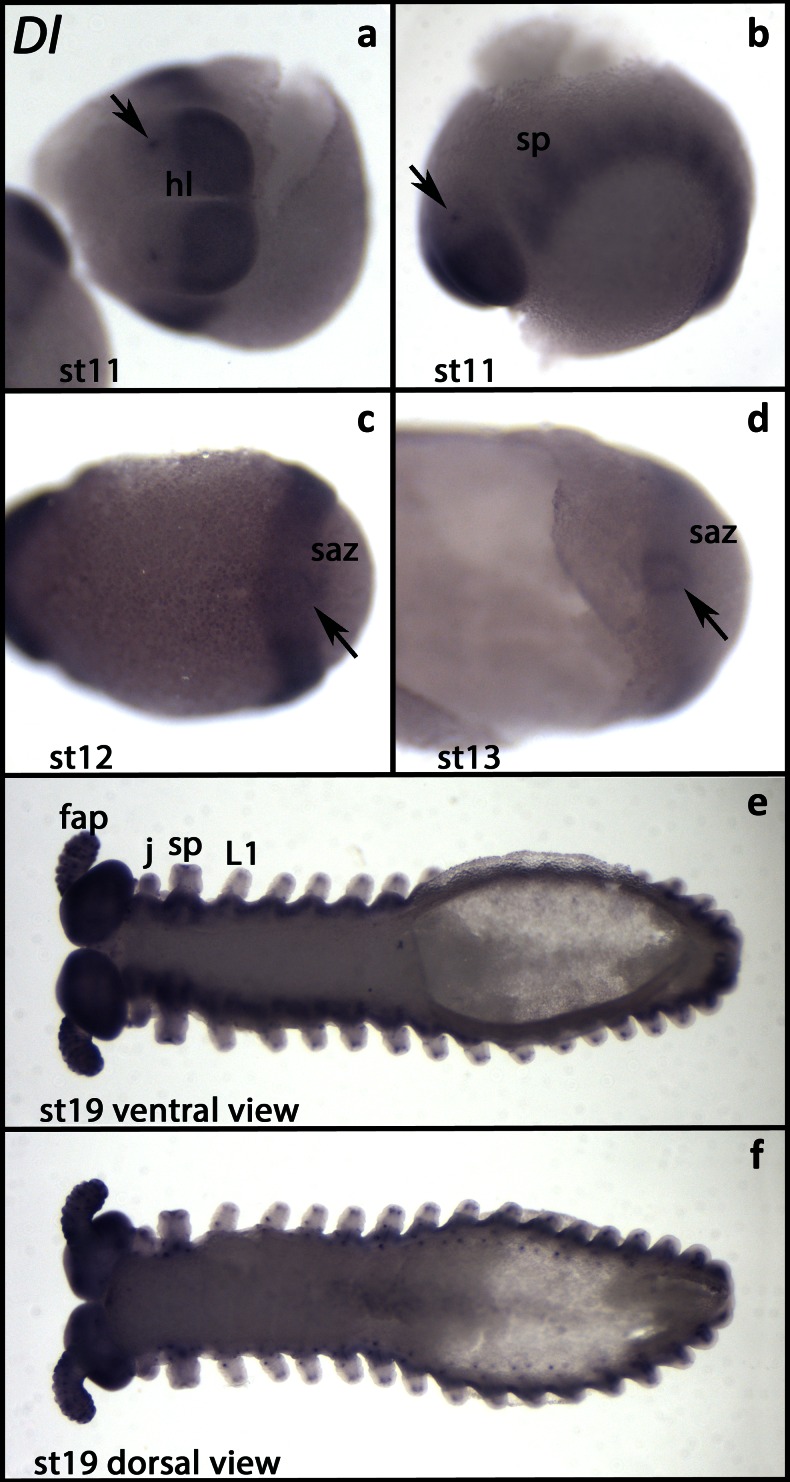
Expression of *Delta*. In all panels, the anterior is to the *left*. **a** View of the anterior of the embryo (stage 11). *Arrow* points to a spot of expression in the primordium of the frontal appendage. **b** The same embryo as in **a** (lateral view). *Arrow* as in **a. c** Ventral view. *Arrow* points to the weak expression surrounding the posterior pit. **d** Posterior part of an embryo (ventral view, stage 13). *Arrow* as in **c. e** Ventral view (stage 19). **f** The same embryo as in **e** (dorsal view). Abbreviations as in Fig. 1

At early stages, all tissues except the saz express *Euperipatoides Delta* (*Ek-Dl*) ubiquitously but at low levels (Fig. S1C). Later, expression disappears from the anterior of the head lobes, exactly as it is the case for *N* (Fig. 2a, b). In the anterior head lobes, only few cells express *Ek-Dl* (Fig. 2a, b). This pattern is also comparable to that of *Ek-N*, and we assume that expression is in the same cells. The segment addition zone does not express *Ek-Dl* (Fig. 2c, d). Like *Ek-N*, also *Ek-Dl* is expressed in the posterior pit, but the domain of *Ek-Dl* is smaller and expression is weaker than that of *Ek-N* (Fig. 2c, d). At later developmental stages, *Ek-Dl* is expressed ubiquitously in tissue ventral to the limbs but is upregulated in two distinct domains per segment (Fig. 2e). Comparable expression has been reported for *Dl* in *E. rowelli* (Oliveira et al. 2013). In the limbs and in tissue dorsal to the limbs, *Ek-Dl* is expressed in single cells or small cell clusters (Fig. 2f). We assume that this expression is correlated with the development of sensory organs (e.g. Walker and Tait 2004).

**Fig. 3:**
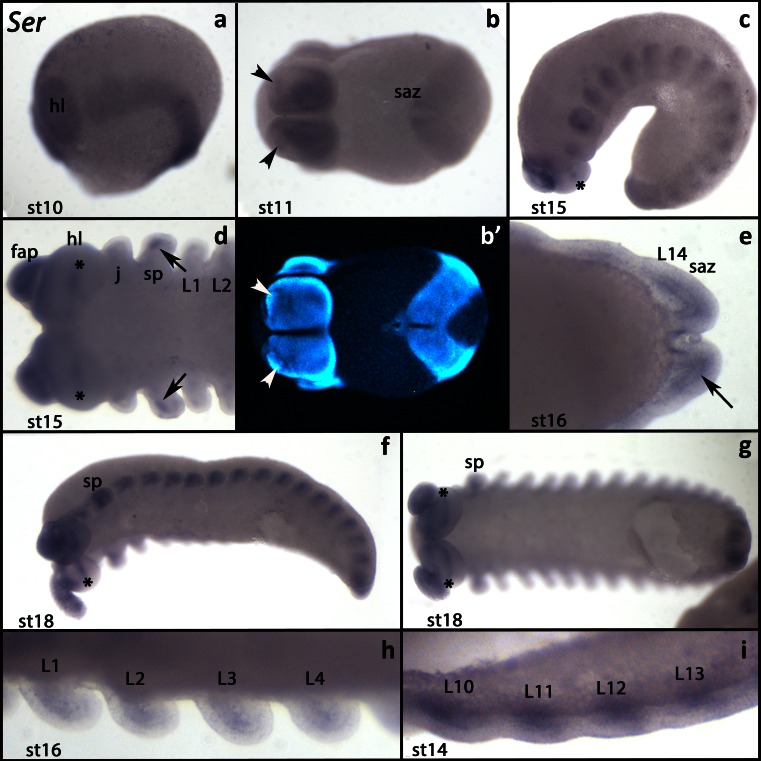
Expression of *Serrate*. In all panels, the anterior is to the *left*. **a** Lateral view (stage 10). **b** Ventral view (stage 11). *Arrowheads* mark the expression inside the frontal appendages. **b′** A DAPI counterstaining of the same embryo as shown in **b. c** Lateral view (stage 15). *Asterisk* marks the expression inside the head lobe. **d** Anterior of an embryo (ventral view, stage 15). *Arrows* mark the expression in the mesoderm of the slime papillae. **e** Posterior end of an embryo (stage 16). *Arrow* points to the expression in the mesoderm of the segment addition zone. **f** Latero-ventral view (stage 18). *Asterisk* as in **c. g** The same embryo as in **f** (ventral view). *Asterisks* as in **c. h** Ventral view (stage 16). Close up on walking limbs. Expression in the anterior mesoderm. **i** Ventral view (stage 14). Close up on walking limb bearing segments. Expression in the mesoderm. Abbreviations as in Fig. 1

At early developmental stages, *Euperipatoides Serrate* (*Ek-Ser*) is expressed in all tissues, except the segment addition zone (Fig. 3a). At stage 11, expression in the head lobes becomes restricted to wedge-shaped domains covering the ventral and posterior regions of the head lobes (Fig. 3b). A few cells in the frontal appendages express *Ek-Ser* (Fig. 3b). Later, it is expressed in the anterior mesoderm of the limb rudiments and the growing limb buds and inside the head lobes (Fig. 3c, d, f–i). Throughout development, *Ek-Ser* is weakly expressed around the edges of the posterior pit (Fig. 3b, e).

**Fig. 4:**
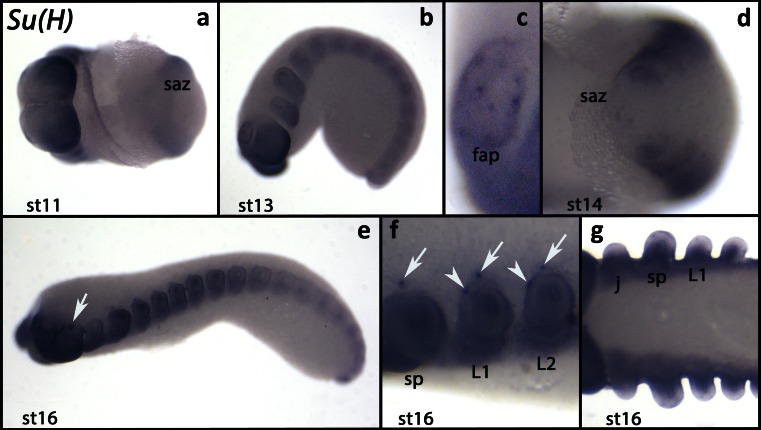
Expression of *Suppressor of Hairless*. In all panels, the anterior is to the *left*. **a** Ventral view (stage 11). **b** Lateral view (stage 13). **c** Close up on a frontal appendage of the embryo shown in **b. d** Close up on the posterior end of an embryo (ventral view, stage 14). **e** Lateral view (stage 16). *Arrow* points to the expression in the eye. **f** Close up of the embryo shown in **e** (lateral view). *Arrows* point to the dot-like expression dorsal to the limbs. *Arrowheads* mark the dot-like expression in the walking limbs. **g** Ventral view. Note that the tips of the limbs do not express *Su(H)*. Abbreviations as in Fig. 1

In early developmental stages, *Suppressor of Hairless* (*Ek-Su(H)*) is weakly expressed in all tissues except the segment addition zone (Figs. 4a, d and S1D). Expression is stronger in the posterior and dorsal regions of the head lobes (Fig. 4a) and in some cells in the frontal appendages (Figs. 4b, c and S2). At later developmental stages, the level of expression increases as the segments mature (Fig. 4e). At this point, a single cell within the ectoderm of each walking limb expresses *Ek-Su(H)* as well as a cell dorsal to the base of the slime papillae and the walking limbs (Fig. 4e, f). Expression in the distal region of the limbs disappears (Fig. 4g).

## Discussion

### Expression patterns suggest that Dl/N signalling is not involved in posterior segment addition in the onychophoran

#### No oscillation

Three animal phyla classically possess a segmented body (but see also Budd 2001; Scholtz 2002 for discussion of what the meaning of segmentation actually is). These are the vertebrates, the annelids and the arthropods including their close relatives, the onychophorans. In vertebrates, a molecular oscillator functions in posterior segment addition. A characteristic of this mechanism is the dynamic expression of a number of genes in the presomatic mesoderm (the vertebrate segment/somite addition zone) (reviewed in Kageyama et al. 2012). Similar oscillators have long been predicted for arthropods as well, where segmentation genes often show dynamic expression in the segment addition zone. Very recent studies in the beetle *Tribolium castaneum* finally provided proof that the detected dynamic expression patterns are the result of oscillation, not cell movement (Sarrazin et al. 2012; El-Sherif et al. 2012). Dynamic expression of segmentation gene orthologs in other arthropods implies that this feature, and thus an oscillating mechanism, is conserved in arthropod segmentation (Chipman et al. 2004; Damen et al. 2000, 2005; Chipman and Akam 2008; Janssen et al. 2011a; Kadner and Stollewerk 2004; Pueyo et al. 2008; Eriksson et al. 2013).

Previously, we have analysed a number of onychophoran segmentation gene orthologs, including those genes that cycle in *Tribolium*, i.e. *odd-skipped* and *even-skipped*. None of these genes show any dynamic expression during segment addition in onychophorans (Janssen and Budd 2013).

The analysis of Dl/N signalling genes in this study further supports the hypothesis that dynamic gene expression is not present in onychophorans, suggesting that at least partially different mechanisms are used to build the segmented body of arthropods and onychophorans.

#### No stripes

Typically, so-called *segmentation genes* are, if not expressed in dynamic patterns in the posterior embryo, either expressed ubiquitously or in transverse stripes in the posterior segment addition zone or they are expressed in transverse stripes in newly formed (or forming) segments or somites. The same holds true for the components of the Dl/N signalling cascade in animals in which this pathway is (likely) involved in posterior segment addition, such as arthropods, annelids and vertebrates (e.g. Reaume et al. 1992; Palmeirim et al. 1998; Stollewerk et al. 2003; Janssen 2005; Schoppmeier and Damen 2005b; Chipman and Akam 2008; Eriksson et al. 2013; Rivera et al. 2005; Thamm and Seaver 2008).

We find, however, that *N*, *Dl*, *Ser* or *Su(H)* are expressed neither in the anterior of the segment addition zone nor in transverse stripes in newly forming segments. Therefore, we conclude that the core of Dl/N signalling is not involved in onychophoran segmentation.

#### No downstream patterning

Pair-rule gene orthologs (PRGs) function downstream of (or level with the) Dl/N signalling in arthropods as shown for a spider (Chelicerata) and a water flea (Crustacea) (Stollewerk et al. 2003; Schoppmeier and Damen 2005b; Eriksson et al. 2013) and as suggested by dynamic gene expression in a variety of arthropods (Damen et al. 2000, 2005; Dove and Stollewerk 2003; Kadner and Stollewerk 2004; Janssen 2005, 2011; Pueyo et al. 2008; Chipman and Akam 2008; Janssen et al. 2011a, 2012; Eriksson et al. 2013).

The situation in vertebrates is similar in that Dl/N signalling is coupled to *hairy*-related genes (Davis and Turner 2001; Kageyama et al. 2012). In *Drosophila*, *hairy* acts as a primary PRG and thus plays an important role in the segmentation process. The function of *hairy*-related genes is likely conserved in other arthropods as well (Damen et al. 2000; Pueyo et al. 2008; Chipman and Akam 2008; Janssen et al. 2011a; Eriksson et al. 2013; but see Choe et al. 2006; Aranda et al. 2008 for studies that suggest that *hairy*-related genes have no (or have lost their) function in trunk segmentation).

We have previously investigated the expression of three *hairy*-related genes (*hairy/Hes*, *Hes2* and *Hes3*) in *E. kanangrensis*. Neither of these genes nor any other pair-rule gene ortholog is likely involved in the segmentation process, since neither of these genes, except *even-skipped* (*eve*), is expressed in the segment addition zone (Janssen and Budd 2013). These data further collaborate with our hypothesis that Dl/N signalling is not part of the onychophoran segmentation process.

### Posterior elongation vs posterior segment addition

Posterior segment addition is correlated with the elongation of the anterior-posterior axis. Recent work on this topic has revealed a number of conserved genetic factors such as *brachyury* (*bra*), *even-skipped* (*eve*), *caudal* (*cad*), the *Wnt* genes and *Dl/N* signalling genes that are involved in the posterior elongation in bilaterian animals (van den Akker et al. 2002; Lohnes 2003; Copf et al. 2004; Chawengsaksophak et al. 2004; Shimizu et al. 2005; de Rosa et al. 2005, Beermann et al. 2011; Martin and Kimelman 2008, 2009; Mito et al. 2011; Williams et al. 2012; Chesebro et al. 2012).

The conserved expression patterns of onychophoran *bra* (Janssen et al. 2015b), *eve* (Janssen and Budd 2013), *cad* (Janssen and Budd 2013; Janssen et al. 2015b) and expression of some *Wnt* genes such as *wg/Wnt1*, *Wnt5* and *Wnt11* as indicators of Wnt signalling (Eriksson et al. 2009; Hogvall et al. 2014) strongly imply that these factors also play a role in the posterior elongation in onychophorans.

Despite the fact that *Dl/N* signalling genes are not expressed in a segmentation gene-like fashion (discussed above), we find that at least *N*, *Dl* and *Ser* are indeed expressed in the posterior pit region at the very posterior pole of the developing onychophoran embryo. This implies that these genes, and thus Dl/N signalling, are likely involved in the posterior elongation in onychophorans as well.

### What controls conserved segmental patterns of segment polarity genes and pairberry (pby) in onychophorans?

In *Drosophila*, the segment polarity genes (SPGs) and the tertiary PRG *paired* (*prd*) are under control of upstream acting PRGs. The PRGs are under control of the gap genes, which in turn are under control of *inter alia*, the posterior determinant *caudal* (*cad*) (reviewed in Pankratz and Jäckle 1993).

Despite the different modes of development in most other arthropods than *Drosophila* (long-germ vs short-germ development), the role of *cad* as a posterior determinant appears to be conserved (Shinmyo et al. 2005; Olesnicky et al. 2006; Nakao 2012; Copf et al. 2003, 2004) as well as the function of SPGs (Ingham 1991; Janssen et al. 2004; Simonnet et al. 2004; Farzana and Brown 2008; O’Donnell and Jockusch 2010). Gene expression pattern analysis and some functional studies also imply that PRGs, and here especially the primary PRGs, are generally involved in arthropod segment addition and likely work level with the Dl/N signalling (e.g. Damen et al. 2000, 2005; Schoppmeier and Damen 2005a; Choe et al. 2006; Choe and Brown 2007; Chipman and Akam 2008; Janssen et al. 2011a). Gap gene-based trunk segmentation likely evolved within the insect lineage (Peel and Akam 2003; see additional text in the supplementary section for more information). In the beetle *Tribolium*, an insect with the less-derived short-germ mode of development, for example, the function of the gap genes is less dominant than in *Drosophila* (reviewed in Jaeger 2011), and here, PRGs are partially under direct control of *cad* (El-Sherif et al. 2014).

In onychophorans, gene expression analysis suggests that the primary PRGs as identified in *Drosophila* and *Tribolium* (i.e. *even-skipped* (*eve*), *runt* (*run*), *hairy* (*h*) and *odd-skipped* (*odd*)) are not directly involved in the regulation of the highly conserved segment polarity gene network (Janssen and Budd 2013), and neither is the Dl/N pathway, as demonstrated in the current article.

**Fig. 5:**
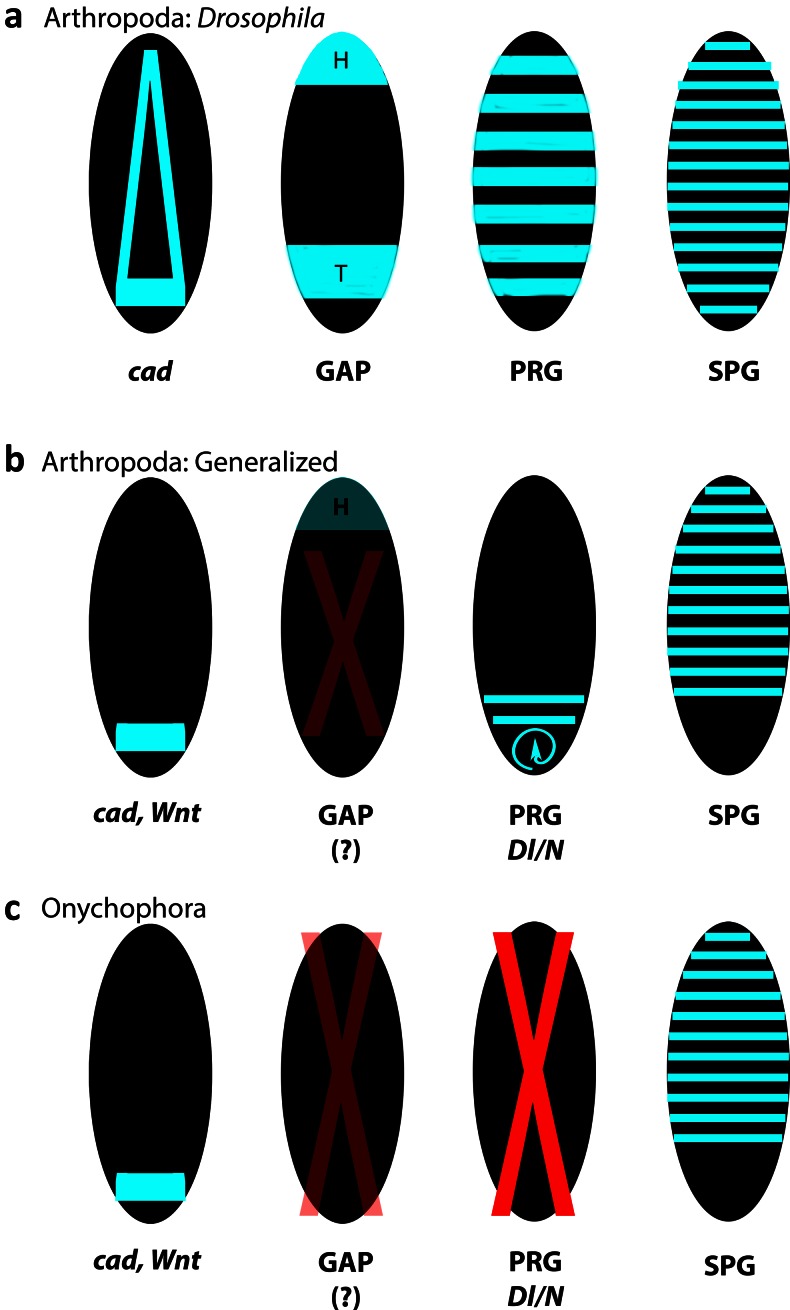
Evolution of segmentation gene systems in onychophorans and arthropods. In the long-germ insect *Drosophila*, segmentation is under control of the segmentation gene cascade (reviewed in Pankratz and Jäckle 1990). The gap gene system likely evolved in the lineage leading to *Drosophila* (see supplementary text for further information). In onychophorans, neither the gap gene system (see supplementary text for further information) nor the downstream acting pair-rule gene (*PRG*) system is conserved. Dynamic expression of Dl/N signalling genes is not present either. It is thus unclear how exactly the conserved patterns of the segment polarity genes (*SPGs*) are established in onychophorans. Possibly, SPGs are under direct control of the conserved posterior patterning system (*cad*, *Wnt*). *Black ovals* represent embryos. Gene expression is in *light blue. Red X* indicates the loss of function during segmentation. *Red X with decreased opacity* indicates a somewhat unclear situation (in Arthropoda) and an incomplete data set (in Onychophora). *Blue circular arrow* indicates the dynamic gene expression in the segment addition zone. *cad caudal*, *Dl/N Delta/Notch* signalling genes, *GAP* gap genes, *H* head, *PRG* pair-rule genes, *SPG* segment polarity genes, *T* trunk

Based on the accumulated gene expression data, we therefore suggest that the posterior elongation system and its likely conserved components (e.g. *cad*, *Wnt* signalling (Chesebro et al. 2012)) may be in direct control of *pby* and the segment polarity gene network in onychophorans and that PRG/Dl/N-mediated segmentation may have evolved in the arthropod lineage (Fig. 5).

In order to test this hypothesis, however, it would be important to establish functional methods to analyse gene function in onychophorans, which are currently lacking.

## Electronic supplementary material

Below is the link to the electronic supplementary material.

ESM 1 (DOC 59 kb)

ESM 2 (XLSX 36 kb)

Fig. S1 Early expression of *Notch*, *Delta* and *Suppressor of Hairless*. In all panels anterior is to the left. A Expression of *Notch*; stage 5; ventral view. Ubiquitous expression. Enhanced expression in the posterior pit. Low signal in newly formed segments. B Expression of *Notch*; stage 8; lateral view. C Expression of *Delta*; stage 8; ventral view. D Expression of *Suppressor of Hairless*; stage 7; ventral view. B’ and D’: DAPI-stained embryos as shown in B and D. Abbreviations: hl, head lobe; pp, posterior pit. (GIF 72 kb)

High resolution image (TIF 24745 kb)

Fig. S2 Early expression of *Suppressor of Hairless in the frontal appendages*. Anterior is to the left. View on to the head lobes; Stage 10. Arrowheads mark expression inside the outgrowing frontal appendages. Abbreviations: hl, head lobes; m, mouth. (GIF 150 kb)

High resolution image (TIF 7322 kb)
