# Supplementary material for: Gene expression analysis reveals that Delta/Notch signalling is not involved in onychophoran segmentation
Source: Dev Genes Evol. 2016 Mar 2;226:69–77. doi: 10.1007/s00427-016-0529-4 (PMC4819559; doi:10.1007/s00427-016-0529-4)
Supplement: Supplementary file 1 — (DOC 59 kb) [file 427_2016_529_MOESM1_ESM.doc]

Supplementary data:

Additional text.

*The gap gene system in arthropods and onychophorans*

Data on ´classical´ *Drosophila* trunk gap genes such as *knirps* (*kni*), *Krüppel* (*Kr*), *giant* (*gt*) and *hunchback* (*hb*) outside the insects are sparse, most probably because their function in segmentation is not (or only little) conserved. One exception may be represented by *hb* that acts as a gap gene in a spider (Schwager et al. 2009) and that is expressed in a gap gene-like domain in the developing head of a myriapod (Janssen et al. 2011a).

To complicate the matter, genes that do not function as gap genes in *Drosophila* can have such a (or a related) function in other arthropods. One example is the gap gene-like function of the pair-rule gene *even-skipped* in a cricket (Mito et al. 2007). Another example is the gap gene-like function of the spider gene *Disal-less* (*Dll*) (Pechmann et al. 2011), a gene that is otherwise known as a key-regulator of limb development (Panganiban and Rubenstein 2002). It is thus unclear if a gap gene-system existed in the last common ancestor of the arthropods, and if, how this system looked like and what factors where initially involved. Therefore, making a general statement about gap gene function in Arthropoda is difficult (cf. Fig. 5B).

A certain level of conservation is seen in the anterior gap gene-like patterning system in at least mandibulate arthropods. Some of the so-called head gap genes like *orthodenticle* (*otd*), *buttonhead* (*btd*), *collier* (*col*), and again *hb*, are at least expressed in comparable patterns in mandibulate arthropods (e.g. Economou and Telford 2009, Birkan et al. 2011, Janssen et al. 2011b, Hunnekuhl and Akam 2014, Sharma et al. 2014), although functional data show that their function may be quite diverse (Schinko et al. 2008, Schaeper et al. 2010). Nevertheless, a conserved set of genes may ancestrally be involved in head patterning in arthropods; in such a scenario the situation in chelicerates would be derived. Therefore, it is difficult to make a general statement about the presence/conservation of an ancestral arthropod anterior gap gene-based patterning system (cf. Fig. 5B)

Data on onychophoran trunk gap gene orthologs are restricted to the analysis of *hb* in the onychophoran *Euperipatoides rowelli* (Franke and Mayer 2015). The authors show that *hb* is not expressed in gap gene-like patterns throughout segmentation. Gene expression data on a *knirps*-like (*eagle*-like) and *Krüppel* orthologs, that are present in the sequenced embryonic transcriptome (RJ, unpublished data), are not available yet. The anterior (head) patterning system as known from *Drosophila* and other arthropods is not conserved either in onychophorans although some of these genes may have a function in head development (Steinmetz et al. 2010; Janssen et al. 2011; RJ, unpublished data). One reason for that may be that all segments form from a posterior segment addition zone in onychophorans, and not from the blastoderm as the anterior segments in arthropods.

References used in the additional text:

Birkan M, Schaeper ND, Chipman AD (2011) Early patterning and blastodermal fate map of the head in the milkweed bug *Oncopeltus fasciatus*. Evol Dev 13:436-447

Economou AD, Telford MJ (2009) Comparative gene expression in the heads of *Drosophila* melanogaster and *Tribolium castaneum* and the segmental affinity of the *Drosophila* hypopharyngeal lobes. Evol Dev 11:88-96

Franke FA, Mayer G (2015) Expression study of the *hunchback* ortholog in embryos of the onychophoran *Euperipatoides rowelli*. Dev Genes Evol 225:207-219

Hunnekuhl VS, Akam M (2014) An anterior medial cell population with an apical-organ-like transcriptional profile that pioneers the central nervous system in the centipede *Strigamia maritima*. Dev Biol 396:136-149

Janssen R, Budd GE, Damen WG (2011a) Gene expression suggests conserved mechanisms patterning the heads of insects and myriapods. Dev Biol 357:64-72

Janssen R, Damen WG, Budd GE (2011b) Expression of *collier* in the premandibular segment of myriapods: support for the traditional Atelocerata concept or a case of convergence? BMC Evol Biol 11:50

Mito T, Kobayashi C, Sarashina I, Zhang H, Shinahara W, Miyawaki K, Shinmyo Y, Ohuchi H, Noji S (2007) even-skipped has gap-like, pair-rule-like, and segmental functions in the cricket *Gryllus bimaculatus*, a basal, intermediate germ insect (Orthoptera). Dev Biol 303:202-213

Panganiban G, Rubenstein JL (2002) Developmental functions of the Distal-less/Dlx homeobox genes. Development 129:4371-4386

Pechmann M, Khadjeh S, Turetzek N, McGregor AP, Damen WG, Prpic NM (2011) Novel function of Distal-less as a gap gene during spider segmentation. PLoS Genet 7:e1002342

Schaeper ND, Pechmann M, Damen WG, Prpic NM, Wimmer EA (2010) Evolutionary plasticity of collier function in head development of diverse arthropods. Dev Biol 344:363-376

Schinko JB, Kreuzer N, Offen N, Posnien N, Wimmer EA, Bucher G (2008) Divergent functions of orthodenticle, empty spiracles and buttonhead in early head patterning of the beetle *Tribolium castaneum* (Coleoptera). Dev Biol 317:600-613

Schwager EE, Pechmann M, Feitosa NM, McGregor AP, Damen WG (2009) Hunchback functions as a segmentation gene in the spider *Achaearanea tepidariorum*. Curr Biol 19:1333-1340

Sharma PP, Gupta T, Schwager EE, Wheeler WC, Extavour CG (2014) Subdivision of arthropod *cap-n-collar* expression domains is restricted to Mandibulata. Evodevo 5:3

Steinmetz PR, Urbach R, Posnien N, Eriksson J, Kostyuchenko RP, Brena C, Guy K, Akam M, Bucher G, Arendt D (2010) Six3 demarcates the anterior-most developing brain region in bilaterian animals. Evodevo 1:14

Additional Table

Gene-specific primers and fragment length

Additional Figures

Fig.S1 Early expression of *Notch*, *Delta* and *Supressor of Hairless*

In all panels anterior is to the left. A Expression of *Notch*; stage 5; ventral view. Ubiquitous expression. Enhanced expression in the posterior pit. Low signal in newly formed segments. B Expression of *Notch*; stage 8; lateral view. C Expression of *Delta*; stage 8; ventral view. D Expression of *Supressor of Hairless*; stage 7; ventral view. B´ and D´: DAPI-stained embryos as shown in B and D. Abbreviations: hl, head lobe; pp, posterior pit.

Fig.S2 Early expression of *Supressor of Hairless in the frontal appendages*

Anterior is to the left. View on to the head lobes; Stage 10. Arrowheads mark expression inside the outgrowing frontal appendages. Abbreviations: hl, head lobes; m, mouth.
