# Supplementary figures and images for: Gene expression analysis reveals that Delta/Notch signalling is not involved in onychophoran segmentation
Source: Dev Genes Evol. 2016 Mar 2;226:69–77. doi: 10.1007/s00427-016-0529-4 (PMC4819559; doi:10.1007/s00427-016-0529-4)

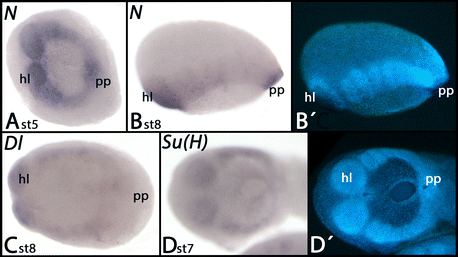

Supplement: Supplementary file 3 — Early expression of Notch, Delta and Suppressor of Hairless. In all panels anterior is to the left. A Expression of Notch; stage 5; ventral view. Ubiquitous expression. Enhanced expression in the posterior pit. Low signal in newly formed segments. B Expression of Notch; stage 8; lateral view. C Expression of Delta; stage 8; ventral view. D Expression of Suppressor of Hairless; stage 7; ventral view. B’ and D’: DAPI-stained embryos as shown in B and D. Abbreviations: hl, head lobe; pp, posterior pit. (GIF 72 kb) [file 427_2016_529_Fig6_ESM.gif]

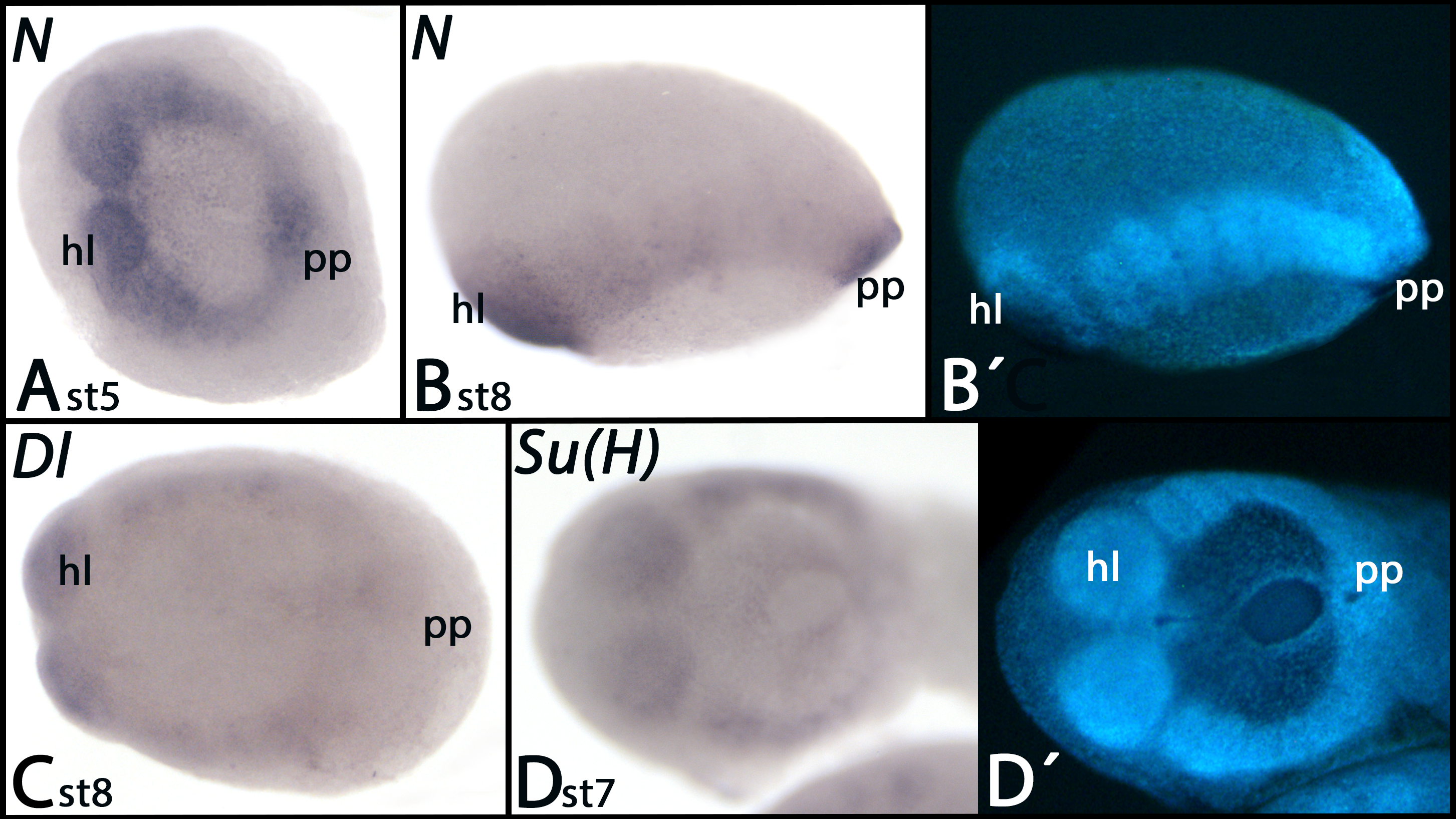

Supplement: Supplementary file 4 — High resolution image (TIF 24745 kb) [file 427_2016_529_MOESM3_ESM.tif]

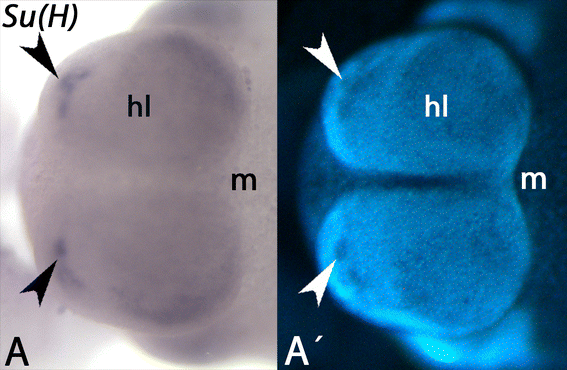

Supplement: Supplementary file 5 — Early expression of Suppressor of Hairless in the frontal appendages. Anterior is to the left. View on to the head lobes; Stage 10. Arrowheads mark expression inside the outgrowing frontal appendages. Abbreviations: hl, head lobes; m, mouth. (GIF 150 kb) [file 427_2016_529_Fig7_ESM.gif]

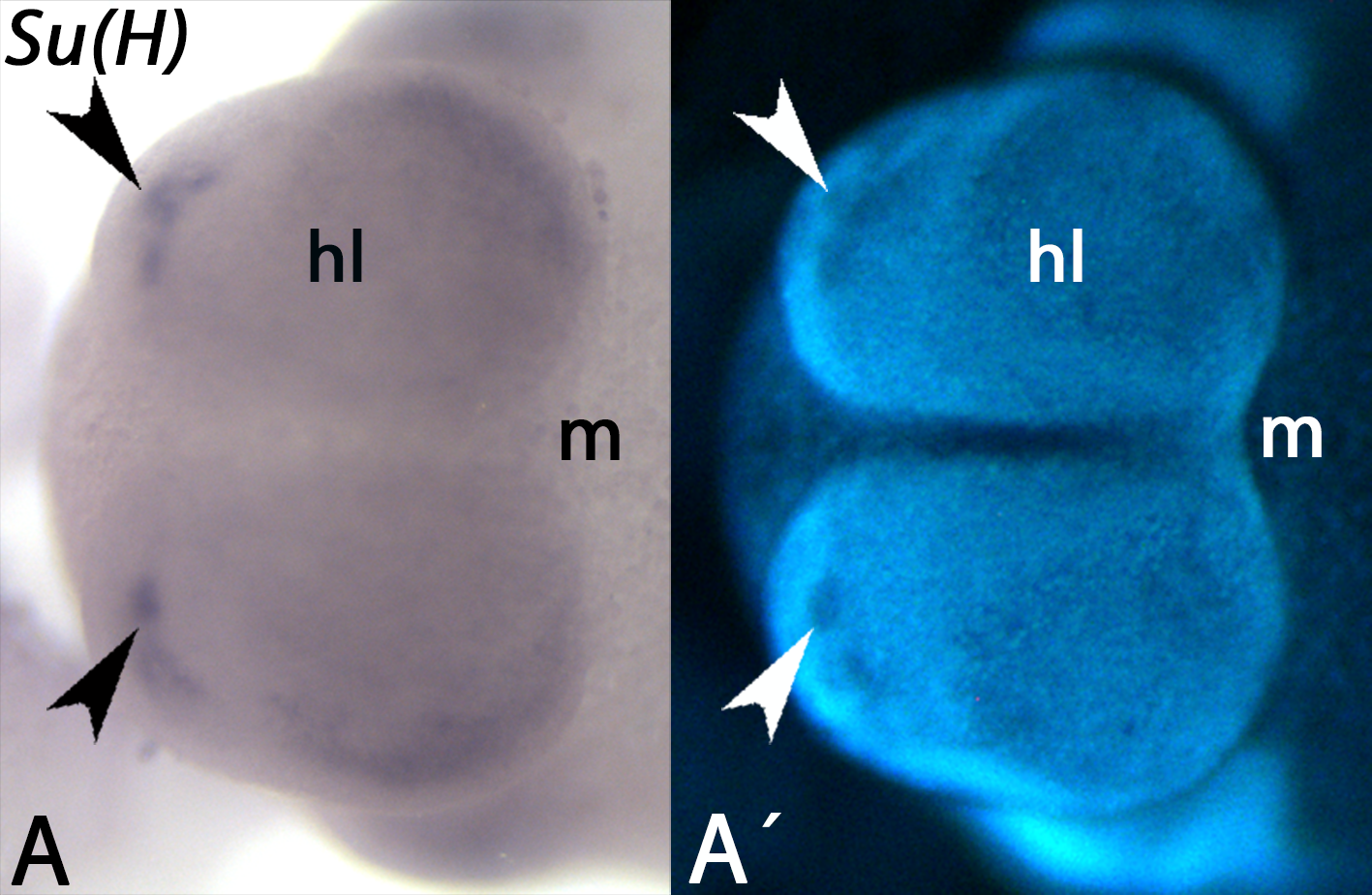

Supplement: Supplementary file 6 — High resolution image (TIF 7322 kb) [file 427_2016_529_MOESM4_ESM.tif]
